# The socio-economic challenges of managing pathogen evolution in agriculture

**DOI:** 10.1098/rstb.2022.0012

**Published:** 2023-03-27

**Authors:** A. G. Geffersa, J. J. Burdon, S. Macfadyen, P. H. Thrall, S. J. Sprague, L. G. Barrett

**Affiliations:** ^1^ CSIRO Agriculture and Food, GPO Box 1700, Canberra, ACT 2601, Australia; ^2^ 1 Catalano Street, Wright, ACT 2611, Australia

**Keywords:** evolution, economics, pathogen, agriculture, integrated disease management

## Abstract

Genetic resistance forms the foundation of infectious disease management in crops. However, rapid pathogen evolution is causing the breakdown of resistance and threatening disease control. Recent research efforts have identified strategies for resistance gene deployment that aim to disrupt pathogen adaptation and prevent breakdown. To date, there has been limited practical uptake of such strategies. In this paper, we focus on the socio-economic challenges associated with translating applied evolutionary research into scientifically informed management strategies to control pathogen adaptation. We develop a conceptual framework for the economic valuation of resistance and demonstrate that in addition to various direct benefits, resistance delivers considerable indirect and non-market value to farmers and society. Incentives for stakeholders to engage in stewardship strategies are complicated by the uncertain timeframes associated with evolutionary processes, difficulties in assigning ownership rights to genetic resources and lack of governance. These interacting biological, socio-economic and institutional complexities suggest that resistance breakdown should be viewed as a wicked problem, with often conflicting imperatives among stakeholders and no simple cause or solution. Promoting the uptake of scientific research outcomes that address complex issues in sustainable crop disease management will require a mix of education, incentives, legislation and social change.

This article is part of the theme issue ‘Infectious disease ecology and evolution in a changing world’.

## 1. Introduction

All agricultural crops are at risk from severe damage by microbial pathogens [1–3]. Tools and strategies to protect crops from pathogen damage are therefore necessary to maintain productivity and food quality. In the field, disease outcomes are heavily influenced by the bio-physical structure of the farming system. In particular, the choice of crop species and varieties, their spatial arrangement in the landscape and temporal sequencing all have the potential to strongly influence epidemiology—as bio-physical complexity increases, disease risk tends to decrease [4]. In this regard, one negative consequence of modern industrial farming is the physical and biological homogenization of landscapes. Farms (and fields) are on average becoming larger, less biodiverse and more spatially uniform [1,5,6]. Such physical and biological uniformity creates ideal conditions for microbial pathogens to rapidly adapt, invade and proliferate [4,7].

Protection for crops against pathogens may be provided by a range of mechanisms, including chemical pesticides, biological control and plant genetic resistance. Among these available tools, genetic resistance to pathogen infection is one of the most valuable. Genetic resistance is an umbrella term for a range of innate plant responses that prevent or reduce pathogen infection and damage. For example, resistance can be a quantitative or qualitative trait, may be partial or complete, and may be conferred by a multitude of different loci or a single gene. Moreover, some genes confer broad-spectrum resistance, while others confer resistance to specific pathogen strains ([8]; for related reviews see [9,10]). Compared to alternatives, resistance is valuable because it requires the lowest level of ongoing inputs, has no harmful environmental effects and is the most cost-effective. However, disease control via genetic resistance is under constant threat from pathogen evolution and resistance breakdown [11–13]. Pathogen adaptation and resistance breakdown often occurs only a few years after resistance is deployed in the field [14], requiring continued efforts to breed new resistant cultivars to restore control [15,16]. Such sequential release of crop varieties typically results in a process akin to an evolutionary arms race, as pathogens adapt in response to the deployment of new sources of resistance. The capacity for plant breeders to respond via the regular release of cultivars with new resistance genes is limited—genetic stocks of novel resistance are finite and are being rapidly depleted [17]. If the rate at which resistance genes are being rendered obsolete in staple food crops is not reduced, there is a very real threat to crop production and food security [18].

Recent research has evaluated the potential for different resistance deployment strategies to increase resistance durability by constraining the evolutionary potential of pathogen populations. These stewardship strategies are based on the targeted use of host genetic diversity to disrupt pathogen evolution, using various combinations of resistance sources deployed over a range of spatial and temporal scales (e.g. pyramiding, cultivar rotations, mixtures and mosaics) [13,19–23]. These strategies work by using resistance gene diversity to variously exploit pathogen fitness costs, functional constraints and life-history constraints, and so disrupt pathogen evolutionary trajectories [11]. While this area of research remains active, results to date consistently demonstrate that engaging in stewardship strategies that incorporate the informed use of resistance diversity in agricultural landscapes can constrain and delay unwanted pathogen evolution. In other words, compared to the *status quo*, strategies for disease management that are explicitly based on knowledge of pathogen evolution have a high likelihood of increasing resistance gene durability [12,19,21,24]. Furthermore, advances in molecular biology and genomics will help enable the engineering of the genetic resources needed to implement such strategies [10], including multi-resistance gene cassettes and isogenic resistance lines. Combined with such technical advances in breeding, the introduction of evolution-based stewardship strategies would promote reliable, long-term disease control, conserve scarce genetic resources and reduce pesticide use [7].

While real benefits will likely be derived from incorporating evolutionary principles into the management of disease in cropping landscapes, there are challenges to their introduction into current management frameworks. One key issue is that while biologists have a good understanding of the problem from an evolutionary and epidemiological perspective, growers and other stakeholders may lack the ability to integrate this perspective into the decision-making process. Critically, we also have limited knowledge of how social, economic and institutional complexities interact to create barriers to the adoption of stewardship strategies [15]. While we focus on these latter complexities below, we do not wish to underestimate the importance of a basic understanding of the eco-evolutionary forces that influence disease dynamics and pathogen evolution.

## 2. Resistance durability: a wicked problem

Given that resistance breakdown has consequences that are costly or impossible to reverse, genes conferring resistance to pathogens should be considered as finite natural resources, thus deserving careful stewardship. Furthermore, durable genetic resistance likely has quantifiable and far-reaching economic and societal benefits. However, while the current evolutionary literature helps guide efforts to manage difficult problems in disease management and resistance durability, there has been little effort to understand the parallel socio-economic dimension. By contrast, significant effort has been invested in understanding socio-economic issues associated with managing the evolution of resistance to antimicrobial compounds [25–29] with consequent development and deployment of resistance management plans (e.g. *one health* [30]). This gap in knowledge for plant resistance means that durable crop resistance is likely undervalued, and incentives for individual decision-makers to invest in managing pathogen evolution are difficult to articulate or support with solid evidence. Embedding genetic resistance and its sustainable use within a defensible economic framework would facilitate a more precise evaluation of strategies and practices that promote resistance durability, thereby exposing potentially attractive incentives for investing in managing pathogen evolution.

However, numerous analytical challenges must be overcome if we are to apply economic principles to better understand the value of genetic resistance as a sustainable disease management strategy. The biological and socio-economic factors that interact over space and time to determine resistance durability exhibit complex inter-dependencies and are thus difficult to predict. This creates a ‘wicked problem’ where there may be conflicting imperatives among different actors, optimal solutions may not exist and incentives for stewardship are consequently difficult to identify (box 1). Given such a wicked problem, there is no straightforward, simple approach for resolving the interacting social, economic and biological uncertainties and complexities that decrease incentives for actions intended to promote stewardship. To help guide future directions, in the following sections, we proceed by exploring the (i) economic value of the genetic resource (i.e. resistance to pathogens); (ii) economic value of managing pathogen evolution and preserving genes that confer crop resistance; and (iii) institutional and political factors that influence the implementation of effective incentive design policies.

Box 1. **Understanding plant genetic resistance management as a ‘wicked problem’.**

While real benefits are likely to be derived from the evolutionary management of disease in cropping landscapes, there are many barriers to the uptake of stewardship strategies that extend beyond the scientific domain. The ‘wicked problem’ concept, taken from sociology, is a useful way to classify and frame complex problems with multi-faceted causes and without clear solutions, encompassing differing perspectives and values by stakeholders [31]. Wicked problems are characterized by the elusiveness of a final resolution, no definitive test for a solution and no generalizable solution that fits all cases [32].

Although the concept of wicked problems arose more than three decades ago, it has recently received growing attention in scientific discourse, as purely technical solutions are increasingly proving insufficient for solving critical societal problems [31,33]. While the concept of wicked problems can be applied to many disciplines, there is growing interest in using this concept to frame problems with underlying evolutionary causes, including pesticide [34], herbicide [33,35] and antibiotic [36,37] resistance.

Resistance breakdown is a clear example of a wicked problem. Some reasons include (i) *Scientific uncertainty*: evolutionary outcomes are stochastic and will inevitably vary depending on the nature of the specific crop–pathogen interaction and human decision-making (e.g. farmer decisions on variety choice) [13]. Precise outcomes associated with different management strategies are thus very difficult to predict (and hence to assign value); (ii) *Management complexity:* resistance stewardship strategies will likely be more complex (and costly) than the *status quo*. For example, breeders might need to adopt new genetic technologies, increase their repertoire of cultivar diversity and provide management advice to growers. Farmers may require new knowledge and equipment, while adopting approaches that increase spatio-temporal complexity on farms; (iii) *Multiple scales and stakeholders*: evolution and the management of evolutionary interactions take place at landscape scales, meaning that collective action (and education) beyond the scale of single farms will often be required to implement and realize the benefits from a given strategy (area-wide management) [38]; (iv) *Public good nature of genes conferring resistance*: a lack of clearly defined ownership of the resistance gene and the degree of control imposed on its use present challenges in understanding the true economic value of managing pathogen evolution and preserving genes that confer crop resistance. Overall, these interacting social, economic and biological uncertainties and complexities make it difficult to identify incentives for actions intended to promote stewardship.

### (a) The economic value of effective genetic resistance

To determine the conditions under which it is economically beneficial to engage in stewardship and thereby preserve the efficacy of genetic resistance, it is first necessary to quantify the intrinsic value of the biological resources (i.e. resistance genes). Attaching economic values to genetic resistance using economic approaches can be guided by existing economic theories and concepts. In practice, rigorous economic valuation of disease resistance is challenging because genetic resources (including those conferring resistance) typically lie outside formal markets and pricing mechanisms (box 2). In other words, a clearly defined market price for the resource does not exist. Furthermore, assigning value to a single gene is challenging, as the biological and genetic nature of resistance is highly variable and depends on the individual pathosystem (e.g. major versus minor resistance genes).

Economic benefits of genes conferring resistance are often estimated in terms of the returns on investments measured as productivity maintenance (i.e. yield losses avoided) through disease control [39–42]. For example, Smale *et al*. [42] and Marasas *et al*. [40] evaluated the economic benefits of investing in productivity maintenance research for disease-resistant wheat varieties. By quantifying the returns on investment in breeding for race-nonspecific resistance to wheat rust in Mexico over the 1973–2003 period, Smale *et al*. [42] estimate that durable resistance to leaf rust yielded a gross benefit of over US$17 million. For the same period, Marasas *et al*. [40] estimated a benefit:cost ratio of 27 : l with an annual rate of return of 41% directly attributable to breeding leaf-rust-resistant wheat varieties in developing countries. Based on field trials of 176 wheat varieties resistant to fungal pathogens in Germany, Lüttringhaus *et al*. [39] estimated that resistance breeding increased winter wheat gross margins by about €4.87 per ha annually. While such analyses are useful, quantifying the true economic value of genetic resistance remains a challenge because the ability to accurately estimate economic benefits in the context of productivity maintenance is dependent on resistance remaining effective [40,43].

**Figure 1.**
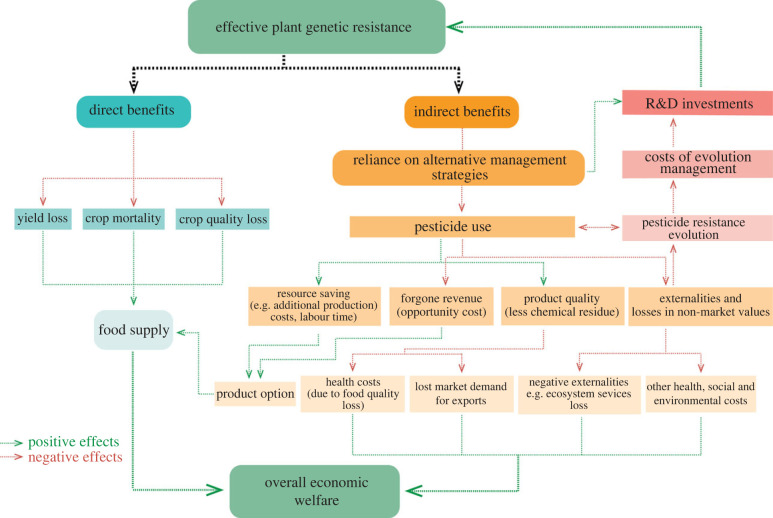
Direct and indirect economic benefits of implementing effective plant genetic resistance. The simplest value of genetic resistance arises from yield gains and reductions in crop mortality and crop quality losses. Indirect benefits can range from saving farm resources to wider benefits which can be broadly categorized as externalities and non-market benefits (e.g. reduced health costs, improved or maintained ecosystem health, and reduced evolution of pesticide resistance). Additionally, the productivity gains resulting from delayed pesticide resistance evolution can significantly benefit stakeholders by lowering investment in evolution management, which in turn generates spillover benefits by freeing resources for R&D investments that would further enhance the effective implementation of plant genetic resistance to pathogens.

In addition to direct effects on yield loss, effective plant genetic resistance can generate other direct and indirect economic benefits, although these are rarely recognized (figure 1). For example, beyond directly reducing yield losses, effective genetic resistance can increase food supply by lowering production costs [44,45]. Effective plant genetic resistance can also impact food supply by avoiding social concerns about reductions in food quality and safety resulting from increased reliance on pesticides [46–48]. Although such indirect non-yield benefits have rarely been considered, they can sometimes even exceed the value of the direct yield benefits [45]. A likely important indirect benefit of effective resistance arises through the reduced reliance on alternative disease control strategies, e.g. pesticides. Because plant genetic resistance is not the only option available to farmers for disease control, clear articulation of the costs and benefits of effective plant genetic resistance, relative to alternatives, is critical for placing value on resistance stewardship practices. For example, using an economic model which accounts for costs of pesticide inputs, Mooney *et al*. [44] have shown that failure to account for input costs spent on alternative disease control options underestimates the benefits of genetic resistance.

**Figure 2.**
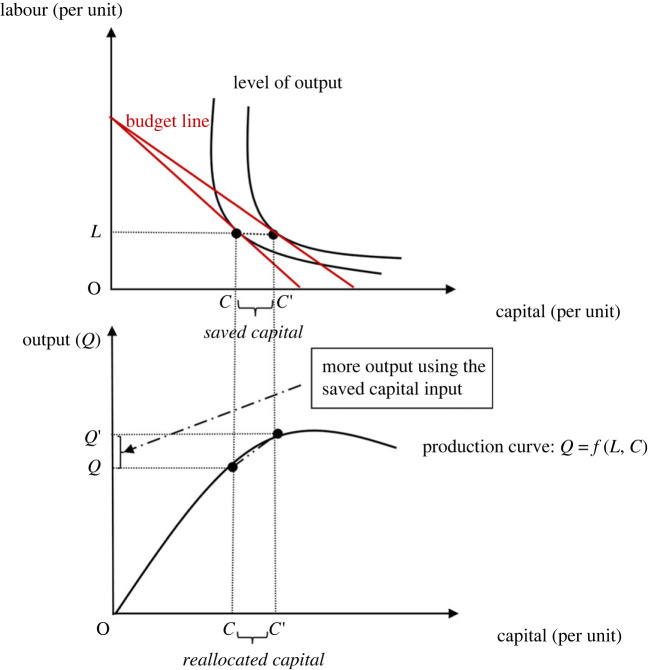
Reallocation of saved capital input. Effective plant genetic resistance can generate resource-saving benefits by freeing scarce resources for other productive on-farm activities. Heavy reliance on capital-intensive alternative disease control strategies (e.g. using pesticides) incurs additional capital costs for the farmer, including the purchase of pesticides and machinery, which could be reallocated to other production activities.

Trade-offs between the alternative allocation of scarce resources play a central role in economic decision-making because they help to distinguish the best alternative. Relative to alternatives (e.g. pesticide application), at the farm level the key relative economic benefit of effective genetic resistance is that it reduces (fully or partially) the requirement for labour and capital inputs (costs of stewardship are discussed below). In other words, effective genetic resistance has resource-saving benefits that result in a reduction in the cost of production [44,45]. For example, disease control using pesticides incurs a range of costs for the farmer, including the purchase of pesticides, labour costs and additional production costs for purchasing and maintaining machinery (capital), which could be reallocated to other production activities (figure 2). This increased ability to divert resources into other activities will likely increase the overall value of adopting strategies that enhance the durability of genetic resistance, but such opportunity costs are rarely accounted for.

Although pesticides play an important role in crop protection by reducing within-season losses due to disease, their indiscriminate use can cause undesirable consequences for the environment, biodiversity and human development by threatening sustainable food production [49–51]. Other alternative disease control strategies (e.g. burning of crop residues), likewise often have undesirable side effects. The indirect benefits of durable plant resistance can therefore be at least partly captured by accounting for the reduction in such negative consequences. Such benefits include, but are not limited to, reducing health costs, improving market demand for export, and improving or maintaining critical ecosystem services. Some of these benefits can be broadly categorized as externalities and non-market benefits (figure 1).

In economics, externalities are defined as the costs (negative externalities) or benefits (positive externalities) that accrue to a third party not directly involved in the economic activity. Agricultural practices such as crop disease management decisions can generate either positive or negative externalities. Genetic resistance to manage plant diseases can likely help mitigate a range of negative externalities that may arise from the heavy use of alternative pathogen control strategies (e.g. fungicides). While studies on plant disease are lacking, such negative externalities have been documented for the chemical control of insect pests. For example, spraying pesticides on one farm can impose a negative externality on adjacent farms by harming beneficial, non-target organisms [52,53]. Pesticide use can also have broader environmental, economic and social consequences via, for example, health and social costs associated with reductions in food quality [46,54]. In the USA, the annual health costs associated with the use of organophosphate pesticides have been estimated to be up to US$44.7 billion [54]. In this regard, effective deployment and management of genetically based plant resistance has strong potential to generate significant economic benefit by minimizing negative externalities resulting from the downstream effects of alternative disease management strategies.

Another major source of negative externality is the development of pesticide resistance [55]. Pathogen resistance to pesticides is now widespread [56], with negative effects on agricultural productivity [57], ecological function and sustainability in farmlands [58]. Where applicable, it may be more valuable to focus research efforts on increasing the durability of genetic resistance to disease than improving our understanding of how to manage pesticide resistance. Implementation of effective resistance gene management strategies has potential benefits, not only by directly contributing to productivity gains, but also ultimately by reducing the ongoing costs of pesticide resistance management. Lowering investment in pesticide resistance management in turn generates spillover benefits by freeing additional resources for investment in research and development (R&D) to further enhance the durability of crop genetic resistance (figure 1). Such potential R&D feedback loops have not previously been investigated or even well articulated.

Overall, positive externalities generated by sustainable resistance management could boost agricultural productivity by reducing pesticide residues, which in turn could contribute to enhancing soil fertility [48,59], increasing soil microbial diversity [60,61] and restricting the evolution of pesticide resistance (so increasing the longevity of alternative management tools). Together, these externalities have the potential to generate substantial non-market benefits to the farming community and society more generally. However, realizing such benefits likely requires mechanisms to encourage the incorporation of externalities into farming decisions and ensure that private benefits and costs reflect the true social benefits and costs. Although not directly reflected in the cash flow of farmers, externalities should ideally be included in the calculation of net profit to fully capture the economic benefits accrued from using genetic resistance. Therefore, accounting for externalities through relevant public incentives and governmental support is likely important to enhance stakeholder attitudes to stewardship.

To fully understand the wider economic benefits arising from effective plant genetic resistance, a comprehensive economic evaluation of the social and individual (farmer) benefits, as well as long- and short-term direct and indirect impacts is needed (figure 1). In commercial agriculture, making an economic valuation of genetic improvements is relatively straightforward because indirect non-yield benefits are often reflected in market price differentials [45]. For example, the economic value of genetic resistance (genetic traits) in genetically engineered *Bt* cotton can be estimated from the market value accrued by breeding companies, as *Bt* technologies are traded directly on the market (see box 3). Unfortunately, in the case of resistance genes, it is difficult to capture indirect economic benefits because they are not typically sold as an input into the breeding process. Given that resistance genes are not market goods, economic models for non-market valuations that integrate choices involving information about the costs and benefits of alternative disease management strategies can be applied to value-specific genes (or traits) that confer resistance. There is a range of economic approaches for non-market valuations with potential application to the valuation of genetic resources at the farm/breeder level (see box 2).

Box 2. **Economic approaches for non-market valuations to value genetic resources.**

In the absence of market prices, one can use economic approaches for non-market valuations to value a genetic resource of interest (e.g. resistance genes). Stated preference (also known as stated choice) survey-based non-market valuation approaches are potentially useful for valuing genetic resources. Such approaches include the *contingent valuation* method and *discrete choice* modelling, which are widely used to estimate the value of recreational and environmental goods (for a review, see [62]). The *contingent valuation* technique elicits an individual's willingness to pay for the benefits accrued from the existence of the resource or to avoid the possible risk (or loss) a person would feel in the absence of the resource [52,63]. The *discrete choice* approach quantifies the value of a resource by presenting respondents with a series of choice sets (usually three or more alternatives) with a combination of several attributes that capture different values [62].

A recent development in pricing methods for valuing *ecosystem services* and *natural capital* [64,65] also offers good insights on valuing resistance genes as exhaustible natural capital. Another potential method for valuing resistance genes could be the *real options* valuation approach, which helps estimate the potential value of a resource using the willingness of people to purchase the resource at a particular price. The real options analysis has been widely used to value genetically modified traits in new crop technologies (see [66]) and measure economic resilience (e.g. [67]).

### (b) The economic value of evolutionary management and resistance stewardship

The fundamental aim of resistance stewardship is to maintain resistance durability in the face of pathogen evolutionary adaptation. As outlined above, crop traits that confer resistance to disease are likely to confer diverse benefits. However, resistance stewardship strategies may be costly, and uptake will depend at least in part on identifying the circumstances under which it is economically beneficial to undertake stewardship. For example, using resistance gene diversity to disrupt pathogen adaptation requires the introduction of biological complexity into breeding programmes and farm landscapes, which may be costly to develop and maintain.

**Figure 3.**
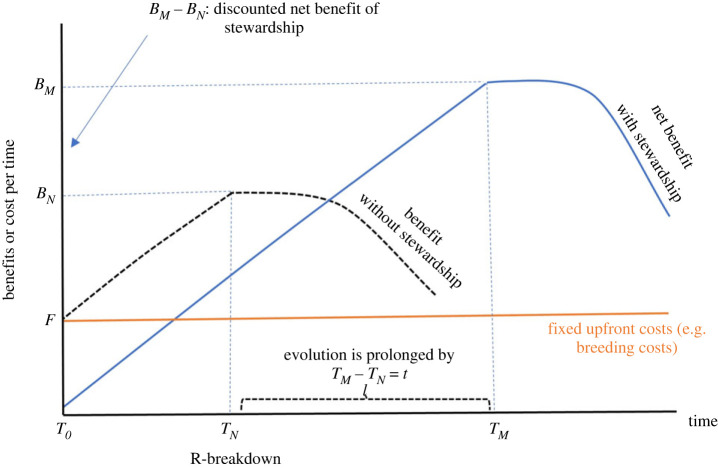
Potential benefits of implementing plant genetic resistance management strategies. Assuming the time evolution occurs is measured in discrete units (e.g. years), the two plots show benefit per unit time in two scenarios (with and without resistance management interventions) where a new source of resistance is deployed in a crop species. Following deployment, resistance genes will have a period (*T*_N_) where they will remain effective, even without management interventions that enhance durability. Although the benefits of management strategies at any given point in time may vary among stakeholders, for ease of illustration, we use a single curve representing best-practise stewardship management. As the adoption of management strategies is not costless, we have two components of costs: costs paid upfront (fixed costs, *F*) and ongoing-management costs, which are reflected in the net benefit. In both scenarios, the net benefit curve first increases but, after evolution evolves, it decreases because adverse evolution reduces the benefits per unit of time. Stewardship management has the benefit of prolonging the time until evolution occurs (by *t* = *T*_M_ − *T*_N_), as compared with a no stewardship scenario.

One of the issues that complicate the economic quantification of resistance stewardship relates to the temporal nature of evolutionary adaptation. It occurs over potentially long periods of time, and while it can be potentially managed, the outcomes of interventions are subject to considerable stochasticity. What may be an economically optimal choice in the short term will likely differ from the choice that minimizes the probability of resistance breakdown over a longer period. For example, consider two scenarios where a new source of resistance is deployed in a crop species (figure 3). In one scenario there is stewardship, which incurs costs, while in the other there is no stewardship. In either scenario, the net benefits of deployment increase until resistance breaks down. Following deployment (*T*_0_), the resistance gene will have a period (*T*_N_) where it will remain effective, even without stewardship. During this period (*T_N_* − *T*_0_), the costs of stewardship will outweigh the benefits. However, with stewardship, the time until breakdown will be extended (*T*_M_). The difference in lifespan conferred by stewardship (i.e. *t* = *T*_M_ − *T*_N_) represents the additional benefits of planned evolution management, captured by a discounted net benefit (*B*_M_ − *B*_N_). Hence, stewardship practices have the potential to realize long-term value, but this may take many years to be realized and the timeframe is uncertain.

Such time lags between implementing the practice and receiving the benefit generate additional economic complexity. Economic models must be flexible enough to account for this temporal complexity [27]. Cost–benefit analyses, which consider both current and future costs and benefits, can be used to evaluate stewardship strategies. In doing so, it is important to account for trade-offs between current accrued benefits and the costs of lost future effectiveness. This is done in the context of economic discounting, a technique used to express the economic notion that a dollar today is of higher value than a dollar earned in the future [68]. In such analyses, the ‘discount rate’ converts the present value to its future value. Generally, the benefits of emerging technologies accrue over a long timeframe but involve upfront costs. This makes farmers' and breeders' discount rates a key driver of the uptake and application of stewardship principles. The longer the time until benefits can be fully realized, the more stewardship will be constrained by upfront investment costs. On this basis, the adoption of stewardship may be more likely for pathogens with the ability to rapidly evolve to overcome resistance [20], as the benefits will be realized sooner.

The net benefit of management strategies at any given time point will vary among stakeholders, which is also likely to influence the perceived value of investing in stewardship. Achieving mutually beneficial outcomes requires strong partnership and cooperation among breeders, farmers, consumers, industry, researchers and policymakers. Among these different stakeholders, it is therefore critical to identify: (i) who benefits from stewardship; and, most importantly, (ii) do benefits only flow to those that actively engage or is there leakage that allows non-contributors (i.e. ‘cheaters’) to gain an advantage? In this regard, one of the important contributing factors relates to ownership of the resistance gene and the degree of control imposed on its use. Ownership rights are an essential attribute of an economic good, as a lack of clearly defined property rights can cause market failure.

Ownership of an economic good or resource is generally categorized into (i) private goods (rivalry and excludable), (ii) common-pool resources (rivalry and non-excludable) or (iii) public goods (non-rivalrous and non-excludable). The two main characteristics that distinguish the three categories of economic resources are *non-rivalrous* and *non-excludability*. Non-rivalrous means that one party's use of the resource does not draw down the stock available to other users, whereas non-excludability means that one user cannot exclude others from using the resource. The non-rivalrous and non-excludable nature of public goods often poses a ‘free-rider’ problem in its sustainable management because an individual decision-maker does not fully contribute to the maintenance of the resource, yet has unrestricted access to it [69].

Material that falls within the private property category is owned and can be more tightly controlled through intellectual property (IP) and patent measures by the owner. This means the owner has a greater capacity to impose stewardship controls on the use of privately owned genes and minimize pest evolution. For example, in Australia, the successful, long-term, pre-emptive stewardship of engineered *Bt* genes (derived from the bacterium *Bacillus thuringiensis*) in cotton varieties [70–73], which confer resistance to insect pests (most notably *Helicoverpa armigera*), has been at least partly enabled by clearly defined private ownership of the genetic resource (see box 3). However, such control is relatively difficult to implement for genetic resources generated and maintained via public breeding programmes (the vast majority), because they fall in the far less controlled public property arena. In such cases, the use of genetic resources by one farmer or plant breeder does not typically preclude their use by another, so private incentives to implement stewardship strategies are likely low. In particular, the sharing properties of public goods mean that genetic materials conferring resistance lie outside formal markets and pricing mechanisms. This makes ownership a fundamental challenge for accurate quantification of the economic value of resistance stewardship. Because genetic material conferring resistance is not sold as an input into the breeding process, the lack of a market price, which is central for economic evaluations, poses a challenge in capturing the true economic value of resistance stewardship and quantifying the economic benefits of stewardship. Consequently, private incentives to hold and protect the resource are generally lower than their true value to users. This creates difficulty in ensuring the optimal use of resistance genes relative to what would be economically efficient when accounting for the costs of lost future effectiveness.

Box 3. **Biological and socio-economic management of transgenic *Bt* crops.**

The widespread use of toxin genes from *Bacillus thuringiensis* (*Bt*, a soil bacterium), which produces proteins that are toxic to insect species (Lepidoptera), in genetically engineered crops [74], has transformed many agricultural industries (most commonly corn and cotton) since the introduction of *Bt* crops in the 1990s. In an analogous way to genetic resistance, *Bt* protects crops from damage caused by insect pests, maintaining productivity and significantly reducing reliance on pesticides [71,75]. However, like genetic resistance, evolution of resistance to *Bt* threatens the efficacy and longevity of control, and stewardship is essential for long-term durability.

There are few good case studies of resistance gene management from which to draw inference about what influences uptake of stewardship practices. *Bt* transformed crops serve as a rare example of how (i) scientifically informed, responsible stewardship can be successfully used to promote resistance durability, and (ii) socio-economic factors directly influence the on-farm application of stewardship strategies.

The genetic factors encoding *Bt* resistance in insects (typically a homozygous recessive trait) have enabled the design of relatively simple stewardship strategies with demonstrated efficacy for managing unwanted evolution. Refuges of non-*Bt* crops form the foundation of resistance management strategies, by allowing susceptible insects to survive and mate with any surviving resistant insects. Because resistance alleles are rare on emergence and genetically recessive, resistant phenotypes are low in frequency in the subsequent generation and selection for resistance is reduced. When used as recommended, pre-emptive resistance management has been shown to be highly effective at slowing pest evolution and enhancing *Bt* durability, with clearly demonstrated flow-on economic and environmental benefits accruing from reduced pesticide use [73].

An important factor influencing stewardship uptake relates to the transgenic nature of *Bt* crops, which has implications for IP, ownership and regulatory control over the use of engineered varieties [72]. *Bt* provides a relatively straightforward case for valuing specific genes, as these are traded directly on the market. For example, the economic benefits that would accrue via commercial income to breeding/biotechnology companies can be used to value specific genes the cotton contains. In contrast with pathogen resistance genes, which are typically public goods, *Bt* technologies and crop varieties fall within the private property category and are protected by patents. This means that breeders and technology providers have incentives to preserve *Bt* efficacy (e.g. via stipulating the use of stewardship strategies in legally binding contracts with growers) [72]. However, despite the availability of relatively simple and effective management strategies, and clearly defined technology ownership, private incentives for individual growers to maximize short-term profits by reducing investment in stewardship remain, and resistance continues to emerge in many regions [76].

A comparison of the success of resistance management in different jurisdictions shows that outcomes vary widely and suggests that governance structures are critical to the success of *Bt* resistance management. Problems with resistance are clearly worse in regions where stewardship compliance and enforcement are poor. For example, in Australia and the USA, government mandates exist regarding the regulation and monitoring of non-*Bt* refuge requirements, driving high levels of farmer compliance and low levels of *Bt* resistance. By contrast, in countries such as Brazil, South Africa and India, compliance is voluntary, and resistance has evolved rapidly and widely [77]. Hence, policies for the management of evolutionary problems must take regional differences in regulatory enforcement into account.

### (c) Socio-political and institutional aspects

The biological complexity of implementing resistance management strategies at scale and the economic uncertainty surrounding the true value of plant resistance complicate efforts to implement resistance stewardship strategies (box 1). In the absence of transformative technological innovations (i.e. a biotech ‘silver bullet’) or societal change (e.g. banning of fungicides in key jurisdictions), collaborative approaches will be pivotal to the successful implementation of stewardship strategies, with farmers, breeders, researchers and governing bodies all required to play their part. However, different stakeholders will likely have conflicting imperatives; incentives for all stakeholders to engage with stewardship may be difficult to identify. For example, an individual farmer may choose to continuously grow the same crop variety over multiple seasons in order to maximize short-term profits, thus generating evolutionary risks for the broader collective. Alternatively, breeders may be reluctant to bear the costs associated with generating genetic resources required for stewardship of a resource they have no ownership over. These conflicts between individual preferences and collective interests will in turn generate social dilemmas that interfere with the uptake and efficacy of stewardship strategies [78]. Barriers or enabling factors for individual stakeholders need to be identified and used to inform decision-making processes that consider issues beyond the scale of single farms or businesses. This includes the need for educational initiatives to provide practitioners with the ability to integrate a basic understanding of the factors that drive pathogen evolution into management decisions.

The challenge of achieving social and economic goals in the presence of a highly heterogeneous pool of stakeholders is not unique to genetic resistance management (and is a common factor for wicked problems in general). There exists a growing number of case studies that consider conditions that can affect the success of institutional arrangements and collaborative efforts in the context of sustainable agriculture [79–81], pesticide resistance (see [34,35,78]) and climate change [81,82]. Such conditions include both internal characteristics of specific stakeholders (e.g. trust, motivation and environmental values) and external factors (e.g. availability of funding, clarity of the rules and public-good nature of the issue) which are beyond the control of the collaborative initiative [81]. Irrespective of the governance structure, the priority outcome needs to be clearly defined and agreed upon by all stakeholders with support to enable meaningful collaboration between actors to implement management strategies at the appropriate scale [80,81]. For example, in healthcare, the World Health Organization (WHO) released a Global Action Plan in 2015 to combat antibiotic resistance [83]. Since 2017, multi-sectoral collaborative plans, known as *One Health,* have been put in place by the WHO in collaboration with Food and Agriculture Organization and the World Organisation for Animal Health to achieve better public health outcomes [30]. Subsequently, many countries have developed National Action Plans and policies that set stewardship expectations to address the threat of antibiotic resistance [25]. Currently, there is no equivalent for the analogous challenges facing the plant-based agricultural sector.

From a policy and governance perspective, promoting the uptake of research outcomes addressing complex issues in sustainable crop disease management likely requires a mix of incentives, legislation and social change. Conventional policy tools aimed at enhancing the uptake of sustainable disease management practices at the farm level must consider the collective dimensions of the management strategies to be implemented. With regard to pesticide resistance management, current strategies generally rely on educational or technical assistance programmes and often ignore the social, cultural and economic complexities of farm-level decision-making [34,35]. Given the public good nature of genetic resources, relying on education and extension alone is unlikely to change individual behaviour sufficiently to achieve a socially optimal level of use of genetic resources. Therefore, promoting collaborative actions through private and public institutions is needed to drive the effective implementation and regulation of genetic resistance management strategies.

## 3. Conclusion

Plant genetic resistance to pathogen infection remains one of the most valuable tools for crop disease management. However, public ownership of genetic resources, conflicting imperatives of different stakeholders, and the spatial and temporal scales over which biological processes occur, create serious barriers to the uptake of stewardship strategies to prevent resistance breakdown. Promoting the uptake of research outcomes addressing complex issues in sustainable crop disease management will require a mix of education, incentives, legislation and social change. In the absence of regulatory guidelines and policy levers to promote the uptake of stewardship practices, education and the identification of incentives to promote and broaden stakeholder collaboration and engagement are badly needed.

To achieve these goals, collaborative, multi-disciplinary research is required. Theoretical studies have variously explored the benefits of bringing an eco-evolutionary perspective to developing more sustainable approaches to disease management in crop systems, but the existing literature and management models for plant genetic resistance pay little attention to the socio-economic factors that create barriers to the translation of scientific findings. While these biological studies have value, the positive benefits will only be fully realized if a significant effort is also put into the identification of effective incentives for the adoption and uptake of strategies that work in real-world agricultural landscapes.

## Data Availability

This article has no additional data.
